# Special Issue: “Cardiovascular Complications in Renal Diseases”

**DOI:** 10.3390/jcm12165307

**Published:** 2023-08-15

**Authors:** Alexandru Burlacu, Adrian Covic

**Affiliations:** 1 Institute of Cardiovascular Diseases “Prof. Dr. George I.M. Georgescu”, 700503 Iasi, Romania; 2 Faculty of Medicine, University of Medicine and Pharmacy “Grigore T Popa”, 700115 Iasi, Romania; adrian.covic@umfiasi.ro; 3 Nephrology Clinic, Dialysis, and Renal Transplant Center, “C.I. Parhon” University Hospital, 700503 Iasi, Romania

The intricate interplay between cardiovascular (CV) pathology and chronic kidney disease (CKD) encompasses diagnostic protocols (both clinical and paraclinical), outcome assessments (such as mortality, morbidity, and costs), as well as advancements in new therapeutic approaches (including pharmacological, interventional, and surgical modalities). This bidirectional relationship is highly complex. Recent advancements in biomarkers, echocardiographic diagnostic techniques, contrast-free coronary angioplasty procedures, and novel renal denervation methods have opened avenues for modulating outcomes previously unexplored. These breakthroughs hold promise for preserving renal function and enabling the early identification of subclinical CV damage in patients with advanced renal conditions.

This Special Issue from the *Journal of Clinical Medicine* aimed to present the diverse CV implications associated with acute and chronic renal diseases while exploring different facets of diagnosis and the latest advancements in therapeutic strategies. As the coordinators of this issue, we view it as highly significant and captivating, setting the stage for future endeavors such as Issue 2.0 and other forthcoming projects. Its solid impact and potential pave the way for continued exploration and innovation in the field.

The contributors of the seven articles featured in this issue comprise a mix of cardiologists, including clinicians, sonographers, intensivists, interventionists, and nephrologists with expertise in chronic dialysis and renal transplantation. This issue encompasses three articles based on substantial patient cohorts, a narrative review, a meta-analysis, and two additional systematic reviews.

In the setting of CKD, the increased risk of CV mortality cannot be entirely attributed to conventional CV risk factors alone, suggesting a multifactorial etiology. One influential factor implicated in this process is the presence of uraemic toxins, which can impact the structure and function of the arterial system and potentially contribute to the development of CV diseases. In a comprehensive literature review, Foudi et al. [1] described the impact of uraemic toxins on vascular smooth cell function and inflammation/arterial remodeling in the context of CKD. An important observation is that the CV risk in patients with CKD remains elevated, even when traditional CV risk factors are pharmacologically tackled. Therefore, the authors conducted a comprehensive synthesis of ongoing studies focusing on therapies aimed at mitigating systemic inflammation in end-stage kidney disease (ESKD), potentially improving the prognosis of such patients.

A retrospective analysis of a single-center registry database with ESKD patients in chronic hemodialysis who underwent percutaneous coronary interventions (PCIs) explored the debatable cardiovascular benefits of statins during a 10-year follow-up period [2]. The authors inquired whether statin therapy was associated with a reduced risk of adverse CV outcomes (the primary endpoint being CV death). Three significant randomized trials conducted previously, which involved patients undergoing maintenance hemodialysis, did not demonstrate the effectiveness of statins in reducing the risk of cumulative CV events and all-cause mortality, and the current guidelines do not recommend initiating statin therapy for hemodialysis patients. However, it should be noted that these trials did not include a substantial number of patients with both ESKD on dialysis and coronary artery disease (CAD). Consequently, the efficacy of statins in this specific population remains uncertain and warrants further investigation. This study’s findings indicated that statin therapy administered during PCI exhibited a significant association with a reduced risk of subsequent CV and all-cause mortalities, as well as major adverse cardiovascular events, while no impact on non-CV mortalities was observed. Furthermore, after adjusting for various factors such as gender, age, diabetic nephropathy, BMI, beta-blocker usage, high-sensitivity C-reactive protein (hs-CRP) levels, and LDL-C levels, statin therapy was associated with a substantial reduction in the risk of CV death, all-cause death, and MACE.

Prolonged uremia in advanced CKD can lead to subclinical cardiac fibrotic damage. Evaluating myocardial strain using echocardiographic techniques allows for detecting functional changes in the heart muscle within the context of advanced CKD. In addition, specific fibrosis biomarkers such as procollagen type I carboxy-terminal propeptide (PICP), procollagen type III N-terminal peptide (P3NP), and galectin-3 (Gal-3) can be measured to assess paraclinical signs of myocardial fibrosis. The study of Ureche et al. [3] aimed to investigate the relationship between four echocardiographic parameters (ejection fraction (EF), global longitudinal strain (GLS), mean E/e’ ratio, and left atrial volume index (LAVI)) and biomarkers associated with cardiac fibrosis, namely PICP, P3NP, and Gal-3, in patients with ESKD before initiating dialysis. This research revealed a robust correlation between PICP and all four echocardiographic parameters, suggesting that PICP could serve as a potential non-invasive indicator of myocardial fibrosis, impacting both systolic and diastolic dysfunction. However, in this cohort, P3NP and galectin-3 were only found to be associated with ejection fraction, indicating a more limited role in assessing myocardial fibrosis compared to PICP. To the best of our knowledge, this is the first study to evaluate and compare the associations of PICP, P3NP, and Gal-3 biomarkers with echocardiographic measurements in patients with ESKD.

In the meta-analysis published in this section, Brinza et al. [4] evaluated the associations between pre-existing pulmonary hypertension (PH) (documented either by transthoracic echocardiography (TTE) or invasively by cardiac catheterization) and adverse outcomes following kidney transplantation (KT). The primary composite outcome (encompassing mortality and graft-related complications) showed a significant increase in patients with pre-existing PH. Furthermore, pre-existing PH was linked to higher rates of delayed graft function, dysfunction, and failure. The effect remained significant for all outcomes irrespective of PH evaluation, invasively or using TTE. Additionally, patients with pre-existing PH exhibited an elevated mortality risk following KT. These findings support the recommendation for a routine assessment of PH in patients on the waitlist for KT.

Arterial hypertension is a global public health issue, with resistant hypertension presenting a higher risk of cardiovascular events. In a systematic review, Burlacu et al. explored the potential of arterial stiffness as a predictor of response to renal denervation (RDN) in patients with resistant hypertension [5]. Ten studies were included, investigating various markers of arterial stiffness. The results showed that arterial stiffness measurements had a modest to good predictive power for RDN response, with areas under the curve ranging from 0.62 to 0.849. Integration of arterial stiffness with other clinical and paraclinical parameters in multivariate models improved the discriminatory capacity. Arterial stiffness assessment could enhance patient selection for RDN, optimizing treatment outcomes and cost-efficiency. Large randomized clinical trials are needed to validate these findings and inform future guidelines on the use of arterial stiffness in RDN.

The most recent guidelines from the European Society of Cardiology (ESC) have highlighted notable and highly reliable recommendations (class I indication, level of evidence A) regarding reducing iodinated contrast agent usage in PCIs for patients with severe CKD. These recommendations aim to prevent the further deterioration of renal function and subsequent mortality increase. Additionally, these guidelines strongly advocate for using low- or iso-osmolar contrast media at the lowest feasible volume in this specific patient population. In this context, Burlacu and colleagues systematically evaluated the existing evidence on the safety and efficacy of minimum- or zero-contrast intravascular ultrasound (IVUS)-guided PCIs in patients with CKD [6]. The results showed that low-to-zero contrast IVUS-guided PCI was associated with a lower risk of contrast-induced nephropathy and major adverse cardiovascular events compared to conventional PCI.

Furthermore, the use of IVUS guidance allowed for better visualization of the coronary anatomy and improved procedural success rates. Based on these findings, IVUS-guided PCI could be a safe and effective alternative to conventional PCI in CKD patients (especially end-stage kidney disease). However, further studies are needed to confirm these results and to identify specific patient populations or clinical scenarios where this approach may be particularly beneficial.

Given the current context of the SARS-CoV-2 virus pandemic, Genovesi and colleagues published in our section the results of a multicenter study on patients hospitalized with COVID-19 [7]. A cohort of 2816 patients admitted over one year for the COVID-19 disease in two large hospitals was analyzed. This study aimed to examine the impact of CKD on in-hospital mortality and the occurrence of incident atrial fibrillation (AF) in patients infected with SARS-CoV-2. In this extensive population of COVID-19 patients, severe CKD emerged as an independent prognostic factor for in-hospital mortality. Moreover, patients who experienced acute kidney injury (AKI) during their hospital stay faced a twofold increased risk of death. As the estimated glomerular filtration rate (eGFR) declined, the incidence of new-onset AF rose, and it exhibited a significant association with the development of AKI.

In conclusion, this issue, despite its heterogeneous nature and specialized focus, showcases the immense potential of research in the field of cardio-nephrology in enhancing diagnostic methods, prognostic assessment, complication risk evaluation, and the utilization of novel therapeutic approaches for patients with advanced kidney disease and associated cardiovascular complications. It highlights the significant advancements being made to improve patient care and outcomes in this unique patient population.
